# Impaired ACE2 glycosylation and protease activity lowers COVID‐19 susceptibility in Gitelman's and Bartter's syndromes

**DOI:** 10.1111/joim.13426

**Published:** 2021-12-16

**Authors:** Giovanni Bertoldi, Verdiana Ravarotto, Luca Sgarabotto, Paul A. Davis, Laura Gobbi, Lorenzo A. Calò

**Affiliations:** ^1^ Nephrology, Dialysis and Transplantation Unit, Department of Medicine University of Padova Padova Italy; ^2^ Department of Nutrition University of California Davis California USA

Dear Editor,

The SARS‐CoV‐2 pandemic (COVID‐19) has focused attention on the renin angiotensin system (RAS), specifically angiotensin‐converting enzyme 2 (ACE2) as it serves as the entry point of the SARS‐CoV‐2 virus [1]. The SARS‐CoV‐2 virus attaches to its target cell via its surface spike (S) protein binding to ACE2 [2], which is then followed by subsequent fusion of the viral envelop with the host cell membrane through the action of specific proteases, such as cathepsin (Cat)‐L [3]. ACE2, part of the RAS counter‐regulatory system, opposes the activity of the regulatory RAS ACE/angiotensin II/AT1R axis by inducing endothelial‐dependent vasodilation, and anti‐proliferative and anti‐inflammatory effects [4]. However, ACE2's role in the cellular entry of the virus raised clinical concerns regarding increased SARS‐CoV‐2 infection in patients treated with angiotensin receptor blockers or ACE inhibitors that increase ACE2 levels, although it is now clear that ACE2 upregulation has a protective impact on COVID‐19 morbidity and mortality [5, 6].

Gitelman's and Bartter's syndromes (GS/BS), two rare genetic tubulopathies, present with hypokalemia and metabolic alkalosis, high Ang II levels and RAS activation yet normo‐hypotension, protection from cardiovascular and renal remodeling, and—crucially—increased ACE2 and Ang 1–7 levels [7].

During the first Italian wave of the COVID‐19 pandemic in early 2020, we assessed via telephone survey the impact of COVID‐19 on our cohort of 128 GS/BS patients living in the main northern Italy COVID‐19 hotspots. We found that none of them had COVID‐19 symptoms compared to the adjusted northern Italian general population's COVID‐19 prevalence (*p* < 0.008), which again suggests that increased risk of COVID‐19 due to increased ACE2 is unlikely [8]. A second survey on the same cohort 1 year later found that only eight patients tested positive for COVID‐19, of which four were asymptomatic and four had very mild symptoms. Based on this and considering GS/BS patients’ increased ACE2 levels, we sought to investigate the possible factors that render GS/BS patients at a minimum resistant to COVID‐19. Given that blocking ACE2/viral S protein interaction is effective against SARS‐COV‐2 infection and that increased pH, a feature of GS/BS, has been shown to interfere with ACE2 glycosylation (Refs. 1 and 2 in the Supporting Information), we recruited 20 GS/BS patients from the previous survey (13 females, 7 males, 32–68 years), with either GS (*n* = 19) or BS (*n* = 1) and 15 healthy controls (seven females, eight males, 29–52 years) and assessed the levels of mononuclear ACE2 and its glycosylation alongside plasma Cat‐L activity (Supporting Information Methods).

GS/BS patients had higher nonglycosylated ACE2 levels (0.82 ± 0.19 d.u. vs. 0.67 ± 0.13 *p* = 0.01) and lower Cat‐L activity (3.91 ± 1.13 r.f.u. vs. 5.31 ± 0.8 *p* < 0.001) (Fig. 1A,B) compared to healthy subjects. In addition, GS/BS's Cat‐L activity inversely correlated (*p* < 0.001, *r* = 0.78) (Fig. 1C) with blood bicarbonate (HCO_3_^−^), while a negative correlation between ACE2 glycosylated isoform and HCO_3_^−^ approaches statistical significance (*p* = 0.08).

**Fig. 1:**
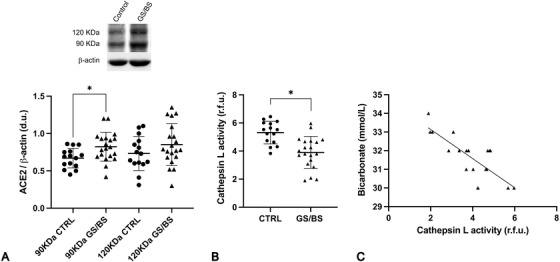
(A) Protein expression of glycosylated (120 kDa) and nonglycosylated (90 kDa) isoforms of ACE2 in Gitelman's and Bartter's syndromes (GS/BS) and healthy control subjects (CTRL). Densitometric analysis of western blot products shows higher expression of nonglycosylated form in GS/BS, compared to controls. Data are shown as mean ± SD. *p = 0.01. (B) Cat‐L levels in GS/BS and CTRL. Cat‐L activity is significantly lower in GS/BS compared to CTRL. Data are shown as mean ± SD. *p < 0.001. (C) Metabolic alkalosis and its relationship with Cat‐L activity. Correlation between blood HCO_3_^–^ and Cat‐L activity showing a significant inverse correlation (*p* < 0.001, *r* = 0.78). GS/B: black triangles; CTRL: black dots.

The genetic defects of GS/BS inducing metabolic alkalosis alter chloride transport. Chloride anion (Cl^−^) is a key factor in cellular homeostasis as changes in intracellular Cl^−^ concentration drive gene and protein expression, post‐translational modification, and intracellular/extracellular pH [9]. Endo‐lysosomal pH plays a critical role for the endocytic uptake of SARS‐CoV‐2. Increased intracellular organelle pH, in fact, interferes with both ACE2 glycosylation and the binding via S protein as observed in experiments with chloroquine (CQ)/hydroxychloroquine (HCQ) (Ref. 1 in the Supporting Information). The inverse correlation in GS/BS between blood HCO_3_^–^ and Cat‐L activity—alongside the trend toward a negative correlation between blood HCO_3_^−^ and the glycosylated isoform of ACE2—also suggests that GS/BS patients’ metabolic alkalosis underlies these effects.

GS/BS's higher level of nonglycosylated ACE2 alongside the reduced Cat‐L activity, which has also been shown to be pH dependent, suggests that the endosomal processing system in GS/BS patients is impaired.

Both glycosylated ACE2 and Cat‐L activity are critical for SARS‐CoV‐2 binding and infection [8]. The increased nonglycosylated ACE2 and decreased Cat‐L activity found in GS/BS patients provide a mechanistic explanation for the near absence of COVID‐19, and the very small number of GS/BS SARS‐CoV‐2 positives found either asymptomatic or with minimal symptoms. In addition, our findings provide a rationale for pursuing the identification and/or synthesis of new drugs that specifically target ACE2 glycosylation and/or proteases involved in SARS‐CoV‐2 infection that avoid the potentially deleterious heart rhythm effects of hydroxychloroquine and chloroquine.

## Ethical Statement

The second survey was a telephone survey as well and the informed consent was asked as reported for the first survey (see ref [8]).

## Conflict of Interest

The authors declare no conflict of interest.

## Supporting information

Supplementary Material and Methods

Supplementary References
