# Supplementary material for: Impaired ACE2 glycosylation and protease activity lowers COVID‐19 susceptibility in Gitelman's and Bartter's syndromes
Source: J Intern Med. 2021 Dec 16;291(4):522–4. doi: 10.1111/joim.13426 (PMC9414342; doi:10.1111/joim.13426)
Supplement: Supplementary file 1 — Supplementary Material and Methods [file JOIM-291-522-s001.docx]

**Supplementary Material and Methods**

**Mononuclear cells preparation**

The blood samples were processed immediately after the collection. Plasma fractions were isolated by centrifugation from 35 mL of EDTA anticoagulated blood and immediately stored at -80˚C for the following procedures. Peripheral blood mononuclear cells were isolated by Lympholyte-H gradient (Cedarlane, Burlington, Canada). Total protein extracts were obtained by cell lysis using an ice-cold buffer (Tris-HCl 20mM, NaCl 150 mM, EDTA 5.0 mM, Niaproof 1.5%, Na_3_VO_4_ 1.0 mM, SDS 0.1%) added with protease inhibitors (Complete Protease Inhibitor Cocktail, Roche Diagnostics, Manheim, Germany). Protein concentration was then evaluated by bicinchoninic acid assay (BCA Protein Assay, Pierce, Rockford, USA).

**Assessment of ACE2 protein expression**

ACE2 profile of protein expression was assessed using western blot analysis. Equal amount of proteins (60 μg) were separated by SDS-PAGE (8% acrylamide gel), transferred onto nitrocellulose membranes (Hybond ECL, Amersham, Uppsala, Sweden) and blocked with BSA (5% in Tween-PBS). Membranes were probed overnight with a primary monoclonal antibody against ACE2 that recognizes a nondisclosed epitope located in the region of ACE2 (aa 631–805) that includes part of the ectodomain (aa 18–740), the transmembrane domain (aa 741–761), and the cytoplasmic domain (aa 762–805) (sc-390851, Santa Cruz Biotechnologies, Santa Cruz, CA, USA).

De-glycosylation experiments have shown that ACE2 can be recognized at 100-90kDa as partial or non-glycosylated ACE2 isoform [1,2]. The antibody used in this study is able to recognize both 90 kDa ACE2 and 120KDa ACE2 isoforms, this latter widely recognized as the glycosylated form [3]. The Amersham ECL™ Rainbow Marker-Full Range (12000-225000 Da, RPN800E, GE Healthcare, Amersham, Uppsala, Sweden) has been loaded as marker of molecular weight.

Specific anti-mouse HRP-conjugated secondary antibody (Amersham Biosciences, Uppsala, Sweden) was added. Membranes were then incubated overnight with a primary monoclonal antibody against b-actin as housekeeping gene (A5441, Sigma Aldrich, St.Louis, MO, USA) and the corresponding anti-mouse HRP-conjugated secondary antibody (Amersham Biosciences, Uppsala, Sweden) was added afterwards. Finally, immunoreactive proteins were visualized with chemiluminescence using SuperSignal WestPico Chemiluminescent Substrate (Pierce, Rockford, USA) at the Amersham Imager 600 (GE Healthcare UK Limited, Buckinghamshire, UK). Protein immunocomplex were evaluated by a PC based densitometric semiquantitative analysis using NIH ImageJ software (NIH, Bethesda, MD, USA) and quantification of targeted proteins were normalized using housekeeping b-actin detected in the same membrane.

**Cathepsin L activity**

Cat-L activity was measured using a commercially available fluorescence-based assay (ab65306, Abcam, Discovery Drive, Cambridge, UK). Briefly, plasma aliquots (50 mL) from patients and controls were incubated on a 96-well plate with a synthetic FR-AFC substrate at 37˚C for 2 hours. A background control and a negative control were also included in the plate. Finally, the free fluorescent AFC released was detected (excitation 400 nm and emission 505 nm) using the EnSight Multimode Plate Reader instrument (PerkinElmer, Waltham, MA, USA)

Cat-L activity has been determined by comparing the relative fluorescence units (r.f.u.) with the r.f.u. level of the negative control sample.

**Bicarbonate blood levels**

Metabolic alkalosis, in terms of bicarbonate blood levels, was assessed through hemogasanalysis using RAPIDPoint® 500 Blood Gas System (Siemens Healthineers, Erlangen, Germany).

**Statistical analysis**

Data are presented as scatter dot plot and expressed as mean ± SD.

The normal distribution of the variables was formally verified beforehand by Shapiro-Wilk test and statistical analysis using parametric unpaired Student t test was performed using GraphPad Prism version 9.0.1 for macOS (GraphPad Software, San Diego California USA, [www.graphpad.com](http://www.graphpad.com)). P -Values at 5% levels or less (p<0.05) were considered significant.

**References**

[1] Vincent MJ, Bergeron E, Benjannet S, Erickson BR, Rollin PE, Ksiazek TG, et al. Chloroquine is a potent inhibitor of SARS coronavirus infection and spread. *Virol J* 2005;**2**.

[2] D’Onofrio N, Scisciola L, Sardu C, Trotta MC, De Feo M, Maiello C, et al. Glycated ACE2 receptor in diabetes: open door for SARS-COV-2 entry in cardiomyocyte. *Cardiovasc Diabetol 2021 201* 2021;**20**:1–16.

[3] Blume C, Jackson CL, Spalluto CM, Legebeke J, Nazlamova L, Conforti F, et al. A novel ACE2 isoform is expressed in human respiratory epithelia and is upregulated in response to interferons and RNA respiratory virus infection. *Nat Genet 2021 532* 2021;**53**:205–14.
