# Supplementary material for: Impaired ACE2 glycosylation and protease activity lowers COVID‐19 susceptibility in Gitelman's and Bartter's syndromes
Source: J Intern Med. 2021 Dec 16;291(4):522–4. doi: 10.1111/joim.13426 (PMC9414342; doi:10.1111/joim.13426)
Supplement: Supplementary file 2 — Supplementary References [file JOIM-291-522-s002.docx]

Supplementary references

1 Vincent MJ, Bergeron E, Benjannet S, Erickson BR, Rollin PE, Ksiazek TG, et al. Chloroquine is a potent inhibitor of SARS coronavirus infection and spread. *Virol J* 2005;**2**.

2 Monteil V, Kwon H, Prado P, Hagelkrüys A, Wimmer R, Stahl M, et al. Inhibition of SARS-CoV-2 Infections in Engineered Human Tissues Using Clinical-Grade Soluble Human ACE2. *Cell* 2020;**181**:905-913.e7.
